# Traditional Mongolian Rhythmical Vibration Therapy for Low Back Pain: Acute Mechanisms and Three-Year Sustainability

**DOI:** 10.3390/healthcare14152357

**Published:** 2026-08-03

**Authors:** Molor Radnaabazar, Tserendagva Dalkh, Odontsetseg Ganbaatar

**Affiliations:** 1 Department of Traditional Medicine, Otoch Manramba University, Ulaanbaatar 13361, Mongolia; 2 Department of Traditional Medicine, Mongolian National University of Medical Science, Ulaanbaatar 14210, Mongolia

**Keywords:** low back pain, rhythmical vibration therapy, myotonometry, muscle stiffness, Traditional Mongolian Medicine, longitudinal study, WHOQOL, Terrenkur

## Abstract

**Highlights:**

**What are the main findings?**

- Manual rhythmical vibration therapy significantly reduced lumbar paraspinal muscle stiffness in patients with low back pain.
- Patient-reported functional disability scores (RMQ) decreased from 13 to 3 following a 10-day intervention.

**What are the implications of the main findings?**

- Traditional manual vibration techniques may offer a more effective reduction in muscle stiffness compared to high-frequency mechanical vibration devices.
- The clinical benefits of this intervention, specifically regarding physical independence and quality of life, can be sustained for at least three years when combined with a walking-based maintenance program.

**Abstract:**

Background/Objectives: Traditional Mongolian Rhythmical Vibration Therapy (RVT) is a manual intervention utilizing low-frequency mechanical oscillations, yet its biomechanical effects lack objective quantification. The present study aimed to evaluate the impact of manual RVT on paraspinal muscle stiffness and its long-term sustainability on the quality of life (QoL) in patients with chronic low back pain (LBP). Methods: To evaluate treatment mechanisms and long-term sustainability, this investigation utilized an acute comparative framework (*n* = 60) alongside a three-year longitudinal observational study design (*n* = 60) using consecutive convenience sampling. To assess biomechanical efficacy, paraspinal stiffness was measured via mytonometry, contrasting manual RVT against mechanical percussive vibration. Additionally, the long-term sustainability of outcomes was evaluated where clinical efficacy was quantified using the Roland-Morris Disability Questionnaire (RMQ) and the WHOQoL instrument, supported by a post-treatment metered walking regimen (Terrenkur). Within- and between-group changes were analyzed using paired and independent *t*-tests. Results: A Manual RVT yielded statistically significant and greater reduction in paraspinal muscle stiffness compared to mechanical vibration (*p* < 0.05). Immediate clinical outcomes revealed significant reductions in RMQ scores, which dropped from 13.33 ± 2.046 to 3. 40 ± 1.522 (*p* < 0.001, Cohen’s d = 4.20). At the three-year follow-up, participants maintained significantly high quality of life scores across physical, psychological, and social domains (*p* < 0.001, effect sizes d > 0.80). Conclusions: Manual RVT is associated with reduced paraspinal muscle stiffness in chronic LBP patients. The integration of this manual therapy with a Terrenkur maintenance regimen appears to support the maintenance of functional and quality of life improvements over a three-year period. However, given the observational design of the study, these outcomes must be interpreted cautiously, and randomized controlled trials are required to establish absolute therapeutic efficacy.

## 1. Introduction

### 1.1. Epidemiology of Low Back Pain

Musculoskeletal disorders, particularly chronic low back pain (LBP), represent a primary global health challenge with significant socioeconomic implications. Recent epidemiological data from the Global Burden of Disease Study 2021 indicates that LBP affects over 577 million people worldwide, remaining the leading cause of years lived with disability [1]. Depending on the chronicity and severity, LBP can manifest through a wide spectrum of symptoms, ranging from localized discomfort and postural deviations to severe functional limitations and significant psychosocial dysfunction [2,3]. Modern Clinical Practice Guidelines (CPGs), such as the widely adopted international consensus guidelines, and recent Systemic Literature Reviews (SLRs) universally emphasize that non-pharmacological, conservative manual and physical therapies should be prioritized as first-line interventions to mitigate pain and prevent secondary functional decline [4,5].

### 1.2. Biomechanical Principles

The progression of chronic LBP often follows a deleterious trajectory from manageable paraspinal hypertonicity to joint-level involvement and neural compression [6]. While modern clinical management often prioritizes pharmacological analgesia and diagnostic imaging, these approaches frequently focus on temporary symptom alleviation rather than addressing the underlying biomechanical disturbances, potentially facilitating the transition to chronicity [7,8].

A key characteristic of chronic low back pain is a change in the stiffness and elasticity of the muscles supporting the spine, specifically the lumbar erector spinae. Chronic muscle tightness, whether induced by sudden injury or prolonged sedentary behavior, fundamentally shifts the biomechanics of the entire kinetic chain [9]. These structural alterations are critical because the stiffness of the paraspinal musculature exerts a direct impact on spinal stability and intervertebral health [10]. Alterations in the anatomical position and biomechanics of the spine, as seen in lumbar disorders, lead to persistent disturbances in spinal alignment and overall neuromuscular control [11,12]. To evaluate these changes objectively, digital myotonometry has emerged as a validated, non-invasive tool that allows for the standardized, reliable quantification of mechanical tissue parameters such as static stiffness [13,14].

### 1.3. Principles of Traditional Therapy

In this context, Traditional Mongolian Rhythmical Vibration Therapy (RVT) is utilized as a manual soft-tissue intervention. Unlike mechanical vibration modalities that provide fixed-frequency high-velocity percussion, manual RVT relies on low-frequency manual oscillations where the practitioner adjusts applied force based on the palpatory feedback of tissue resistance. This intervention is conceptually grounded in the Traditional Mongolian Medicine (TMM) principle of ‘Movement within Movement’. While historically transmitted through oral tradition, this clinical theory was formally codified and standardized in a reference textbook on traditional manual therapy [15]. This soft-tissue approach focuses on applying manual oscillatory forces to target myofascial tissue structures, aiming to down-regulate muscle spindle activity, stimulate slow-adapting mechanoreceptors, and facilitate localized relaxation.

### 1.4. Scientific Rationale for the Study

Despite the historical use of RVT, objective quantification of its direct impact on muscle viscoelastic properties remains limited in the literature. Previous investigations into RVT have evaluated its structural impact on the spine. Radiographic assessments in a separate clinical cohort (*n* = 200) demonstrated improvements in correcting structural spinal deviations and intervertebral compression post intervention [16].

However, a significant gap remains in the broader rehabilitation literature: while the acute biomechanical effects of manual and mechanical therapies are frequently documented, there is a profound lack of long-term tracking data evaluating whether these immediate tissue adjustments align with sustained, multi-year improvements in patient quality of life [7]. Furthermore, the comparative efficacy of manually modulated vibration versus automated mechanical percussion on paraspinal viscoelasticity is unexamined.

To address this knowledge gap, the objectives of this study were to evaluate immediate changes in myotonometric stiffness and long-term quality of life (QoL) in patients diagnosed with chronic LBP before and after a 10-day individualized RVT program. Using a dual-framework design comprising an acute comparative cohort (*n* = 60) and a prospective longitudinal observational study (*n* = 60), we compared the efficacy of manual RVT against automated mechanical vibration devices to determine the role of manual modulation in tissue rebalancing.

The study hypothesis was that the implementation of manual RVT would be associated with significantly greater reduction in lumbar muscle stiffness compared to high-frequency mechanical modalities, and that these immediate physiological adjustments would align with sustained improvements in patient-reported quality of life over a three-year longitudinal observational period.

## 2. Materials and Methods

**Figure 1:**
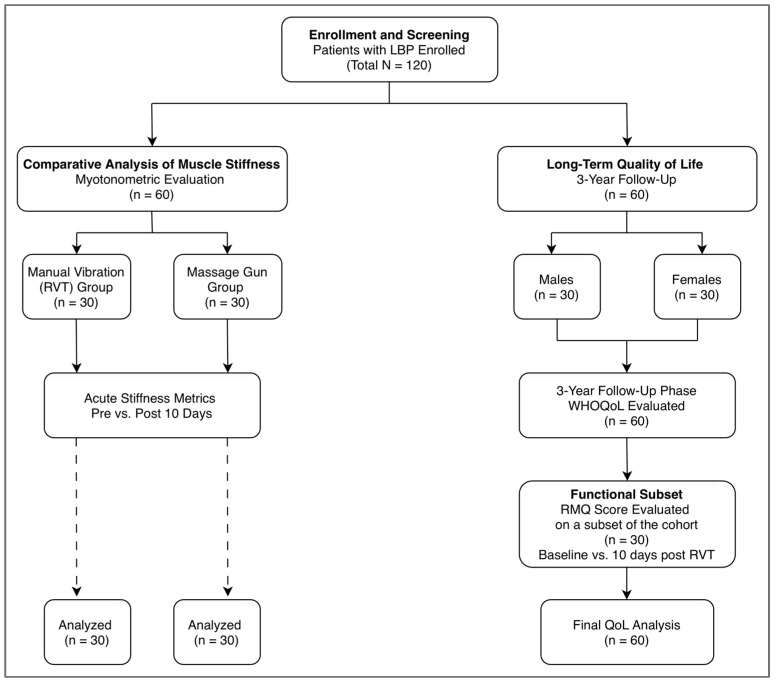
Participant enrollment and follow-up flow diagram adhering to STROBE guidelines.

The study was conducted at Sumadi Hospital from January 2019 to December 2020, followed by a three-year longitudinal assessment. Post-intervention Quality of Life (QoL) data were collected in 2022 and 2023, representing a consistent 36-month follow-up for participants enrolled in the 2019 and 2020 cohorts, respectively. The study framework was organized and reported in accordance with the Strengthening the Reporting of Observational Studies in Epidemiology (STROBE) guidelines (Figure 1). The study protocol was approved by the Ethics Committee of Otoch Manramba University (Approval No. 2019.01) and was performed in accordance with the 1964 Declaration of Helsinki and its later amendments. All participants provided written informed consent prior to enrollment.

### 2.1. Participants

A total of 120 patients diagnosed with chronic low back pain (LBP) were involved in this multi-arm study. Due to the logistics of the clinical setting, participant selection and group allocation were determined via non-randomized, consecutive convenience sampling based on chronological presentation. The analytical structure and exact sample tracking across the study arms are detailed in the STROBE flowchart (Figure 1).

As shown in Figure 1, the total study population comprises two independent clinical arms:

1. The Biomechanical Cohort (*n* = 60): Enrolled strictly for immediate pre- and post- treatment objective myotonometric assessment. These participants were divided equally (n = 30 each) into a manual RVT group and a comparative mechanical vibration therapy.
2. The Longitudinal Cohort (*n* = 60): An independent group consisting entirely of patients who received the 10-day manual RVT course and were monitored for a three-year follow-up period using the WHOQoL instrument (stratified as 30 males and 30 females).

To evaluate short-term clinical functional disability changes without over-surveying, a secondary subgroup represents a non-randomized convenience subset of the longitudinal participants and was concurrently assessed via Roland–Morris Disability Questionnaire (RMQ) at baseline and post-treatment (10 days).

Inclusion criteria for all groups were: (1) age > 18 years; (2) clinical diagnosis of mechanical LBP for at least three months; and (3) radiological evidence of lumbar musculoskeletal involvement. Exclusion criteria included history of spinal surgery, acute inflammatory diseases, malignancy, pregnancy, or current use of muscle relaxants. Comprehensive baseline demographic and anthropometric characteristics (age, sex, and BMI) were captured at the institutional level for the overall parent clinical population from which the present study’s participants were drawn. Overall baseline metrics are provided in Supplementary Materials.

### 2.2. Traditional Mongolian Rhythmical Vibration Therapy (RVT)

The RVT intervention consisted of a 10-day program, with daily 30 min sessions performed by a traditional medicine practitioner with 15 years of professional practice. To ensure the technical consistency of the intervention, the therapy followed standard textbook guidelines [15] and was standardized based on a prior video-kinematic analysis of the lead author’s performance. The technique maintained a rhythmic frequency ranging from 30 to 60 BPM for general myofascial mobilization, with controlled peaks up to 200 BPM for targeted high-density tissue release. This standardized frequency range was applied consistently across the manual group to contrast with the fixed-frequency mechanical percussion.

**Figure 2:**
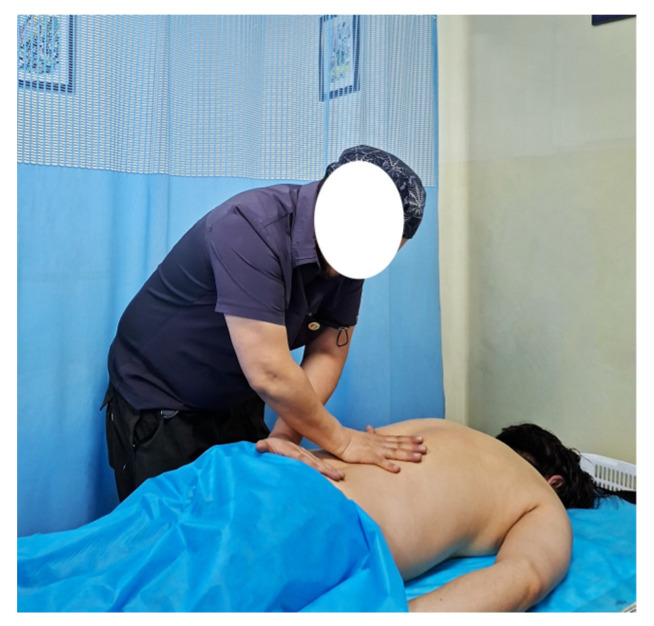
Checking: The practitioner applies gentle pressure along to the spine to locate areas of muscle tightness and stiffness in the lower back.

**Figure 3:**
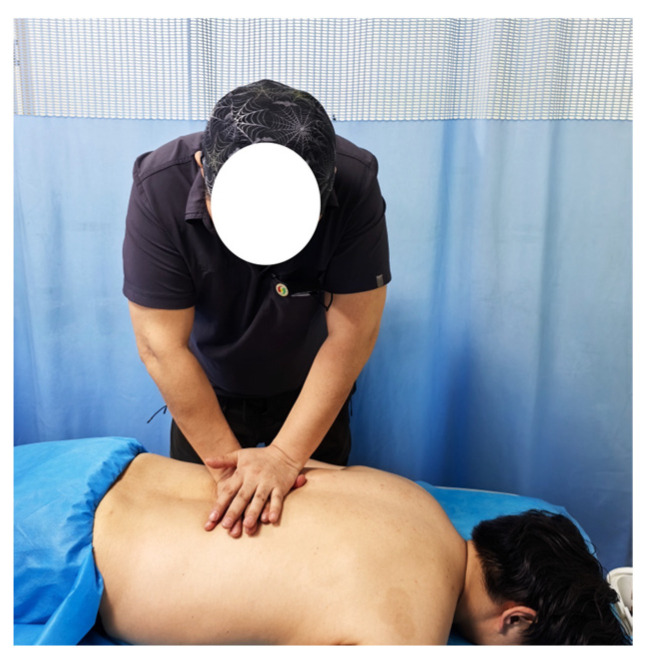
Releasing/Vibrating: The practitioner uses the palm of the hands to apply steady rhythmic vibrations (40–200 BPM) to loosen and relax the tight muscle tissues.

**Figure 4:**
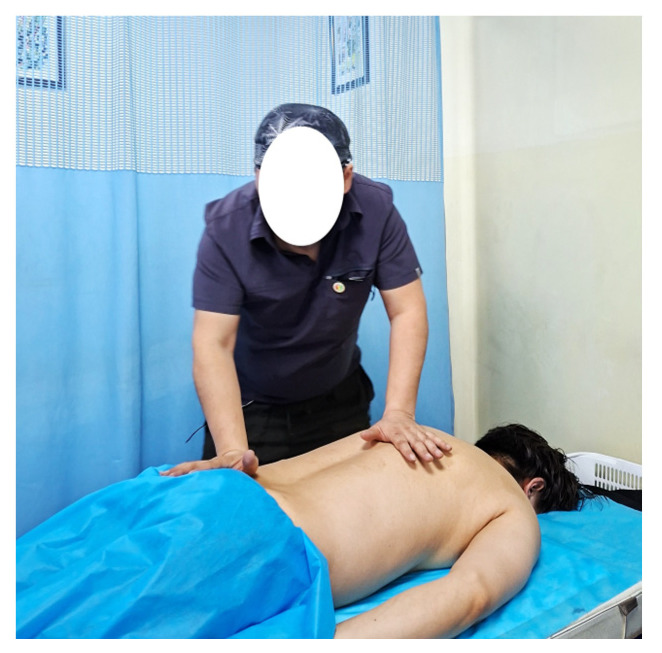
Correcting: The practitioner uses gentle pushing and pulling movements to stretch the lower back and improve spinal alignment.

The manual therapy followed a standardized four-phase clinical cycle:

1. Check (Feeling the muscles): The practitioner uses their hands to feel the patient’s lower back to find tight spots and muscle spasms. As shown in Figure 2, the practitioner presses straight down on the muscles next to the spine to find where the tissue changes from soft and flexible to hard and stiff.
2. Release (Vibrating the muscles): The practitioner vibrates the tight areas using their hands. As shown in Figure 3, the practitioner uses their palm to press down gently, gradually moving deeper while vibrating the muscle between 40 and 200 beats per minute (BPM). The practitioner utilized variable pressure to transition from superficial to deep muscular layers.
3. Correct (Stretching and alignment): Once the muscles are loose and relaxed from the vibrations, the practitioner performs manual adjustments. As shown in Figure 4, the practitioner uses gentle pulling and pushing movement on the lower back to stretch the spine and improve alignment while the muscles are relaxed.
4. Verification (Checking the results): The practitioner tests the lower back one final time to ensure muscles are softer, both sides of the back feel even, and the patient can move more easily.

Note on Video Demonstration: To ensure the RVT method can be easily understood and reproduced by other researchers, a clear, step-by-step video showing the practitioner’s hand positions, vibration speeds, and stretching movements has been recorded. This video will be made open-access and public once the paper is accepted for publication.

### 2.3. Mechanical Vibration (Comparative Group)

**Figure 5:**
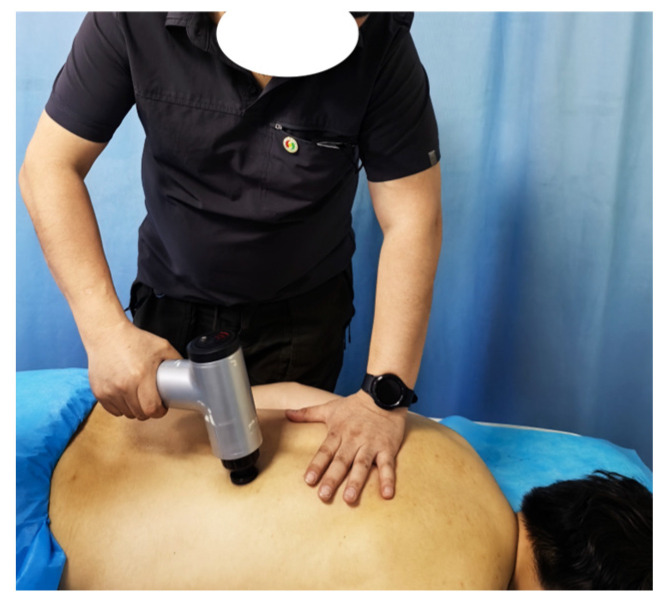
Practitioner applies an automated massage gun to the muscles next to the spine using six different vibration settings.

The comparative group received treatment using a mechanical massage gun GGL KJG-88 (Shenzhen Huayu New Energy Co., Ltd., Shenzhen, China). To ensure a comprehensive mechanical intervention, the protocol involved cycling through 6 pre-set frequency levels (2400–3600 RPM). The device was applied to the same paraspinal regions for a duration equivalent to the manual sessions (Figure 5).

### 2.4. Myotonometric Assessment

Objective measurements of paraspinal muscle properties were conducted using the MyotonPRO device (Myoton AS, Tallinn, Estonia). The device was applied perpendicular to the skin over these paraspinal muscles (L3–L4 level, 3 cm lateral to the spinous processes) while the patient was in a prone position.

Standardized mechanical impulses (force: 0.4 N; duration: 15 ms) were utilized. Due to equipment availability during the study period, the analysis focused on: Dynamic Stiffness (N/m): Measuring the tissue’s resistance to external deformation. Measurements were obtained in sets of five impulses. A coefficient of variation (CV) < 3% was required for data validity.

To ensure absolute consistency across all patients, a single lead practitioner performed every myotonometric assessment. Because the MyotonPRO is a fully automated digital device that generates objective readouts independent of the operator, the risk of measurement bias was inherently minimized.

### 2.5. Post-Treatment Maintenance (Terrenkur)

Following the 10-day clinical phase, a structured Terrenkur (metered walking) protocol was recommended to patients as a home-based program to maintain their progress [17]. The regimen had two phases: an initial Acclimatization phase (2 months of 20–30 min post-prandial walking or 1 km daily) followed by a Maintenance phase. During maintenance, target distances were adjusted based on participant age and BMI to support balance and muscle health (Table 1).

This home program was entirely self-directed by the patients after treatment. Compliance was not formally monitored, and no activity trackers or patient logs were used during the three-year follow up period.

### 2.6. Functional and Quality of Life Outcomes

Functional disability was assessed using the Roland-Morris Disability Questionnaire (RMQ) pre- and post-RVT treatment. The RMQ is a standard 24-item checklist where higher scores indicate greater daily limitations caused by lower back pain.

For the longitudinal analysis, the World Health Organization Quality of Life (WHOQoL) questionnaire was utilized at baseline and at the three-year follow-up mark. This survey evaluated patient well-being across six specific areas (domains):

1. Physical health
2. Bodily image and appearance
3. Level of independence
4. Social relations
5. Financial resources
6. Acquiring new information and skills

Higher scores in these domains represent a better overall quality of life. Both questionnaires were entirely self-administered by the patients to eliminate any potential researcher or assessor bias.

### 2.7. Statistical Analysis

Statistical analysis was performed using IBM SPSS Statistics (version 28.0; IBM Corp., Armonk, NY, USA), supplemented by R (version 4.6.0; R Foundation for Statistical Computing, Vienna, Austria) within the RStudio integrated development environment (version 2026.04.0; Posit Software, PBC, Boston, MA, USA). The normality of the data distribution was assessed using the Shapiro–Wilk test. All data are presented as means ± SD along with their 95% confidence intervals (CI). There were no missing data for the primary outcomes, and only complete participant datasets were analyzed.

To evaluate treatments, the following tests were used:

- Paired-samples *t*-tests were used to compare pre- and post-intervention RMQ scores (*n* = 30), and to compare baseline versus 3-year WHOQoL scores.
- Independent-samples *t*-tests were used to compare the difference between the manual RVT group (*n* = 30) and the mechanical vibration group (*n* = 30).

For all statistical tests, the test statistic (t-value) and exact *p*-values were reported.

To separate statistical significance from real-world clinical relevance, Cohen’s d effect sizes were calculated for all outcomes and interpreted as small (0.20), medium (0.50), or large (>0.80). Because the WHOQoL questionnaire measures six different domains, a Bonferroni correction was applied to adjust the significance threshold for multiple comparisons (0.0083) to prevent false-positive results. For all other single comparisons, the threshold for statistical significance was set at a two-tailed alpha level of *p* < 0.05.

A post hoc power analysis was performed using the dynamic stiffness data from the MyotonPRO device. This analysis indicated that the sample size of 30 patients per group achieved a statistical power greater than 80% to detect true differences between the manual RVT and mechanical massage gun treatments. Similar post hoc evaluations were applied to RMQ and WHOQoL data. However, these retrospective calculations are intended solely to evaluate the statistical power of the completed study and do not substitute for a priori sample size calculation.

## 3. Results

### 3.1. Functional Disability Assessment (RMQ)

A total of 30 participants completed the functional assessment pre- and post-treatment. The results of the Roland-Morris Disability Questionnaire (RMQ) are presented in Table 2. The mean baseline score was 13.33 ± 2.05, which decreased to 3.40 ± 1.52 following the 10-day intervention. This reduction represents a mean difference of 9.73 ± 2.31, which was statistically significant.

### 3.2. Comparative Analysis of Muscle Stiffness (Myotonometry)

Biomechanical assessment of muscle stiffness (N/m) was conducted on a sub-group (*n* = 60) to compare the effects of mechanical vibration (massage gun, *n* = 30) and traditional manual rhythmic vibration (*n* = 30). Both interventions resulted in statistically significant within-group reductions in paraspinal muscle stiffness (*p* < 0.001). The manual rhythmic vibration group exhibited a mean reduction of 13.07 ± 3.18 N/m on the right side and 12.16 ± 3.96 N/m on the left side. The mechanical vibration group showed mean differences of 6.69 ± 3.16 N/m and 6.54 ± 3.31 N/m for the right and left sides, respectively.

**Figure 6:**
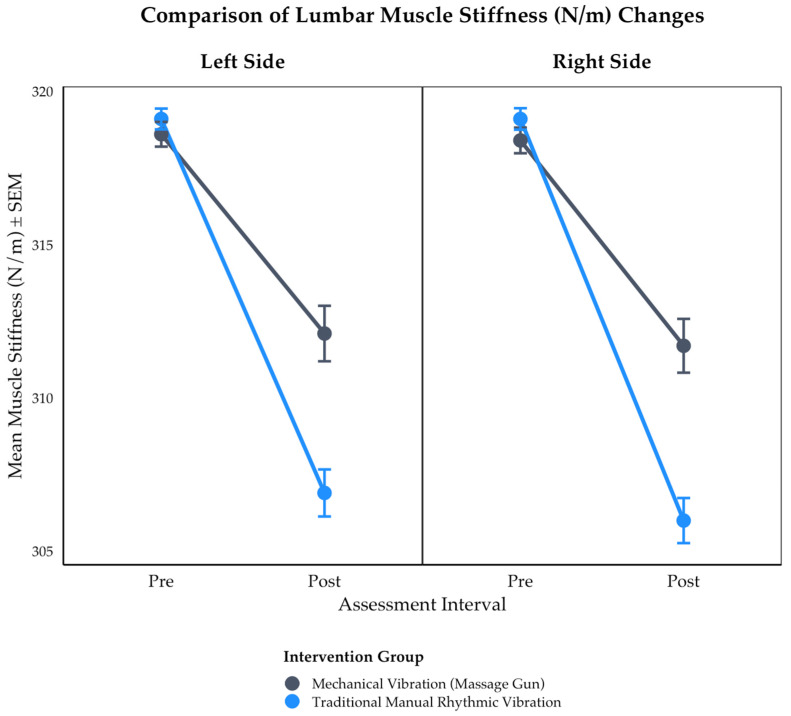
Comparison of bilateral lumbar paraspinal muscle stiffness (N/m) changes between the mechanical vibration (massage gun, *n* = 30) and traditional manual rhythmic vibration (n = 30) groups. Data points represent group means at pre- and post-intervention assessment intervals, and error bars denote the standard error of the mean (±SEM).

Crucially, between-group analysis of individual; change scores revealed that the traditional manual rhythmic vibration group achieved a significantly more pronounced decrease in paraspinal stiffness compared to the mechanical group. On the right side, the manual intervention outperformed mechanical vibration by a net mean difference of 6.38 N/m. This effect was mirrored on the left side, with manual rhythmic vibration providing a mean reduction of 5.62 N/m over the mechanical group. The comparative dynamics of these changes are illustrated in Figure 6, and complete metrics are summarized in Table 3.

### 3.3. Long-Term Quality of Life

#### 3.3.1. Male Sub-Group

The longitudinal impact of the intervention on quality of life was assessed using the WHO Quality of Life (WHOQoL) questionnaire. Results for the male sub-group are summarized in Table 4. Statistical analysis indicated significant increases across all evaluated domains at the three-year post-treatment mark (*p* < 0.001). Notable changes were observed in the Physical Health (PH), reaching 17.10 ± 1.26 from 9.60 ± 2.43 at baseline and Bodily Image & Appearance (BIA), which increased from 9.80 ± 3.08 to 16.60 ± 1.07.

#### 3.3.2. Female Sub-Group

The female sub-group also demonstrated significant changes across all WHOQoL domains three years post-intervention (*p* < 0.001), as detailed in Table 5. Within this group, significant differences were observed in Physical Health (PH), which rose from 10.30 ± 2.76 to 17.30 ± 0.91, and Level of Independence (LOI), which increased from 8.87 ± 2.28 to 15.23 ± 1.43. Bodily Image & Appearance (BIA) domain got a significant improvement, as well.

## 4. Discussion

The findings of this study suggest that Traditional Mongolian Rhythmical Vibration Therapy (RVT) is associated with an immediate reduction in paraspinal stiffness and long-term improvement of quality of life in patients experiencing chronic LBP. However, because this study utilized an observational design without a simultaneous control arm, these variations cannot be definitively attributed to the manual intervention alone. Factors such as the natural history of episodic chronic LBP, spontaneous recovery, or a placebo response must be carefully weighed alongside the specific physical effects of the protocol.

Consequently, the exceptionally large Cohen’s *d* effect sizes observed across all evaluated cohorts must be interpreted with strict caution. Given the modest sample sizes and the lack of a randomized control framework, these values may be inflated by selection bias or natural regression to the mean rather than reflecting pure therapeutic efficacy alone.

### 4.1. Functional Disability Assessment (RMQ)

The drop in RMQ scores from a baseline mean of 13.33 down to 3.40 shows a meaningful shift from marked daily limitations to normal functional tracking. In the context of lumbar rehabilitation literature, a within-patient change of 4 to 5 points is recognized as the threshold for a Minimal Clinically Important Difference (MCID) [18]. Because the improvement in this study (9.93 points) is nearly double the benchmark, the change represents a noticeable practical benefit for the patients. Furthermore, a post-treatment score below 4 points suggests that a significant percentage of the cohort successfully shifted into functional state rather than simply showing statistical change [18].

From a clinical perspective, this rapid recovery may be an essential first step in breaking the cycle of chronic pain. Chronic LBP is frequently prolonged by “protective guarding” and fear-avoidance behaviors, where fear of pain leads to immobilization and subsequent functional withdrawal [19,20]. We hypothesize that the 10-day RVT phase may have acted as a physical catalyst, disrupting this maladaptive cycle. This relief provides acute positive reinforcement, helping patients re-learn that movement does not equal injury [21]. Evidence suggests that while manual therapy provides the initial reduction in stiffness, active movement is required to maintain these viscoelastic improvements over time [22].

While these functional outcomes are promising, the lack of an untreated control group limits our ability to completely separate these improvements from natural healing history of the condition.

### 4.2. Comparative Analysis of Muscle Stiffness (Myotonometry)

The reduction in paraspinal stiffness observed in our myotonometric cohort offers a plausible biomechanical framework for the radiographic improvements in spinal alignment and intervertebral spacing documented in previous literature [16]. These data align with broader clinical research demonstrating that noninvasive myotonometric assessments of passive muscle properties are highly sensitive for tracking musculoskeletal rehabilitation progress and distinguishing pathological hypertonicity from normal tissue characteristics [23]. Historically, the evaluation of traditional manual interventions via objective viscoelastic quantification has been rare. Recent data, however, have shown that myotonometric improvements in muscle tone and stiffness correlate closely with posture correction and pain mitigation in cervical disorders [24]. Our observations extend this reasoning to the lumbar paraspinal region, indicating that resolving deep paraspinal hypertonicity is a significant cofactor in resolving lumbar biomechanics.

A critical finding in this subsection is the observed mechanical discrepancy between manual RVT and mechanical vibration devices (such as massage guns). While high-frequency mechanical percussion (typically 2400–3600 RPM) [25] is widely applied in rehabilitation, high-frequency vibratory stimuli have been shown to trigger the “Tonic Vibratory Reflex” (TVR) via rapid activation of primary muscle spindle endings, resulting in reflexive, protective muscle contraction rather than relaxation [26,27]. Conversely, the lower, bio-resonant frequency of manual RVT (40–200 BPM) is hypothesized to operate below the threshold that triggers a strong TVR. The empirical findings of the present study support this proposed neurophysiological distinction, as between-group analysis revealed that manual RVT achieved over double the stiffness reduction compared to mechanical percussion.

From a neurophysiological perspective, this low-frequency manual input may primarily stimulate slow-adapting mechanoreceptors and interstitial tissue receptors. This suggests a potential tactile pathway to modulate autonomic nervous system activity, theoretically downregulating sympathetic tone and facilitating a localized parasympathetic relaxation response that alleviates active tissue guarding [28]. Furthermore, the steady sliding pressure and rhythmic movement of manual therapy are hypothesized to alter the fluid dynamics within the tissue. This physical manipulation potentially lowers tissue viscosity and relaxes the fascia, making the muscles significantly less rigid [29,30]. Additionally, the practitioner’s manual modulation relies heavily on tactile feedback to sense and adapt to changing tissue tension. This allows for precise, real-time adjustments at the elastic-rigid threshold of the myofascia [15], contrastable with the static application of mechanical tools [31].

### 4.3. Long-Term Quality of Life

The three-year WHOQoL data provide insight into the sustainability of health changes captured across this cohort. In contrast to many standard clinical trials where the functional benefits of short-term manual therapy diminish within 12 months [32], participants of this cohort maintained stable, elevated scores across all domains up to three years post-intervention.

This sustained trajectory highlights a distinct intersection within the biopsychosocial model of chronic pain care. The recovery of basic physical independence likely triggered downstream improvements in social participation and emotional well-being, proving that localized physical relief can shift a patient’s overall lifestyle dynamic [33]. Furthermore, the improvements documented in the ‘Body Image and Appearance’ domain suggest that mitigating asymmetric paraspinal stiffness may have favorably impacted structural symmetry and postural stability [34,35], directly influencing the patient’s physical self-perception and confidence in daily social settings.

Crucially, these long-term gains cannot be credited exclusively to the initial 10-day manual therapy phase. In chronic LBP, resolving local tissue stiffness and dropping functional limitations below the disability threshold (RMQ < 4) alters the baseline movement mechanics of the lumbar region. However, maintaining this baseline requires consistent physical activity to prevent a return to protective muscle guarding. The structured integration of the Terrenkur (walking) regimen during the follow-up period likely acted as the primary vehicle for sustaining these long-term benefits.

Although individual long-term adherence rates to the walking protocol could not be actively monitored, the stability of the three-year post-intervention scores strongly suggests that the cohort maintained a sufficient level of daily physical activity. Regular, low-impact walking facilitates continuous fluid exchange and remodeling within the lumbar extracellular matrix, preventing a return to a hypertonic, protective state.

### 4.4. Strengths, Practical Applications, and Future Lines of Research

A clear strength of this longitudinal design, which tracked a focused patient cohort over a three-year period using both validated patient-reported outcomes (WHOQoL, RMQ) and objective, quantitative myotonometric data. In terms of practical application, manual RVT represents an affordable, non-pharmacological, and accessible treatment modality that can be easily integrated into broader integrative medicine and physical rehabilitation frameworks to provide rapid symptomatic relief and facilitate early mobilization.

However, future research lines must address the methodological limitations inherent in this work. To establish true causal relationships and completely separate the physiological impact of manual, RVT from the placebo effect, natural recovery, or the independent benefits of Terrenkur walking, randomized controlled trials (RCTs) are required. Future protocols should compare manual RVT directly against standardized physiotherapy regimens, mechanical vibration devices, and sham treatments, while implementing rigorous tracking of individual walking adherence during the follow-up phase.

### 4.5. Limitations

First, the lack of randomized controlled design or a true, untreated parallel control group restricts the ability to establish absolute causality. Because participants were not randomly assigned, the influence of the natural history of chronic low back pain (LBP), statistical regression to the mean, or placebo effects on the observed myotonometric and functional improvements cannot be completely ruled out.

Second, the prescription of the Terrenkur (metered walking) regimen introduces a significant confounding variable, particularly regarding the 3-year longitudinal outcomes. While Terrenkur was prescribed uniformly to all participants to support long-term functional status, individual patient adherence to the duration, intensity, and frequency of this walking regimen was not objectively monitored or logged. Consequently, it is impossible to isolate the degree to which the 3-year quality-of-life (QoL) maintenance is attributable strictly to the initial 10-day RVT course versus the sustained, unquantified therapeutic effects of the walking regimen itself.

Third, during the extensive 3-year post-treatment follow-up window (2022–2023), no strict controls or monitoring systems were in place for external lifestyle and clinical variables. Confounding factors, such as intermittent use of analgesics or muscle relaxants, engagement in outside physical exercises, changes in occupational physical demands, or the development of new medical comorbidities, were not documented and may have independently introduced variability into the long-term WHOQoL outcomes.

Fourth, while the core cohorts provided adequate statistical power, the sample sizes remain relatively modest. Crucially, a priori sample size calculation was not performed prior to participant enrollment due to the observational nature of the study design. While the post hoc power analysis demonstrated adequate power (>80%) retrospectively, this does not replace a prospective, a priori justification. Furthermore, the functional disability data (RMQ) was limited to a secondary, non-randomized convenience subsample (*n* = 30), which lacked individual demographic or sex-stratified tracking. This reliance on smaller subgroup evaluations increases the risk of selection bias and limits the capacity to generalize functional recovery trajectories evenly across all participant characteristics.

Finally, this study utilized a single-center framework at Sumadi Hospital, pulling consecutively from a specific clinical population. The unique demographic, environmental, cultural, and physiological characteristics of the studied Mongolian population mean that the baseline tissue properties and lifestyle factors may not align with broader global demographics, thereby limiting the generalizability of these findings to other geographical regions or distinct clinical settings.

## 5. Conclusions

The findings of this observational study suggest that a 10-day Traditional Mongolian Rhythmic Vibration Therapy (RVT) program, paired with a metered walking regimen, is associated with measurable improvements in the biomechanical properties of the lumbar paraspinal muscles. Within this specific cohort, the observed data indicate a notable reduction in muscle stiffness, with manual rhythmic vibration appearing to yield more pronounced improvements in restoring tissue symmetry compared to the evaluated mechanical vibration alternatives. Furthermore, these immediate physiological adjustments correlated with a reduced short-term clinical functional disability and a sustained maintenance of quality of life over a three-year prospective tracking window. However, given the non-randomized, observational design of this secondary analysis, these longitudinal outcomes must be interpreted with caution. Future large-scale randomized controlled trials (RCTs) are necessary to definitely establish the therapeutic efficacy and causal mechanisms of traditional manual therapies against standardized physical therapy protocols.

## Figures and Tables

**Table 1:** Post-Intervention Terrenkur Maintenance Protocol.

Age Group (Years)	Target Daily Distance (km)	Intensity/Phase
Initial (first 2 months)	20–30 min or 1 km	Acclimatization
<30	5.0–8.0 km	Maintenance
31–45	Up to 5.0 km	Maintenance
46–60	Up to 3.0 km	Maintenance
>61	Up to 1.0 km	Maintenance

**Table 2:** Roland–Morris Disability Questionnaire (RMQ) scores before and after RVT.

Assessment Period	Mean ± SD	95% CI	Statistical Analysis
Baseline (Pre-treatment)	13.33 ± 2.046	12.36–13.89	*t*(29) = 22.99
Post-treatment (Day 10)	3.40 ± 1.522	2.83–3.96	*p* < 0.001
Mean Difference	9.73 ± 2.310	8.86–10.59	Cohen’s *d* = 4.20

Paired-samples *t*-test.

**Table 3:** Comparison of lumbar muscle stiffness (N/m) changes between mechanical and manual vibration.

Muscle Side	Intervention Group	Pre-Treatment (Mean ± SD)	Post-Treatment (Mean ± SD)	Within-Group Change (95% CI)	Between-Group Change
Right	Massage Gun	318.4 ± 2.29	311.7 ± 4.81	6.69 (5.51, 7.87)	*t*(58.00) = 7.78 *p* < 0.001
	Manual Vibration	319.1 ± 1.92	306.0 ± 4.02	13.07 (11.88, 14.26)	Mean diff: 6.38 (4.74–8.02)
Left	Massage Gun	318.6 ± 2.21	312.1 ± 4.96	6.54 (5.31, 7.78)	*t*(56.20) = 5.95 *p* < 0.001
	Manual Vibration	319.1 ± 1.86	306.9 ± 4.20	12.16 (10.68, 13.64)	Mean diff: 5.62 (3.73–7.51)

Paired-samples *t*-test for Within-Group; Independent-samples *t*-test for Between-Group.

**Table 4:** Longitudinal WHOQoL Scores for Men 3 Years Post-Treatment.

Domain	Pre-Treatment (Mean ± SD)	3 Years Post-Treatment (Mean ± SD)	Mean Difference (95% CI)	Statistical Analysis
Physical Health (PH)	9.60 ± 2.43	17.10 ± 1.26	7.50 (6.39–8.60)	*t*(29) = 13.84 *p* < 0.001 Cohen’s *d* = 3.93
Bodily Image & Appearance (BIA)	9.80 ± 3.08	16.60 ± 1.07	6.80 (5.67–7.92)	*t*(29) = 12.37 *p* < 0.001 Cohen’s *d* = 2.77
Level of Independence (LOI)	9.27 ± 2.58	14.97 ± 1.29	5.70 (4.70–6.69)	*t*(29) = 11.70 *p* < 0.001 Cohen’s *d* = 2.72
Social Relations (SR)	13.13 ± 1.54	16.67 ± 0.99	3.53 (2.98–4.08)	*t*(29) = 13.08 *p* < 0.001 Cohen’s *d* = 2.64
Financial Resources (FR)	13.40 ± 1.81	15.47 ± 1.10	2.06 (1.50–2.62)	*t*(29) = 7.51 *p* < 0.001 Cohen’s *d* = 1.28
New Info & Skills (OFIS)	16.00 ± 1.55	16.87 ± 1.19	0.86 (0.30–1.42)	*t*(29) = 3.15 *p* < 0.001 Cohen’s *d* = 0.61

Paired-samples *t*-test.

**Table 5:** Longitudinal WHOQOL Scores for Women 3 Years Post-Treatment.

Domain	Pre-Treatment (Mean ± SD)	3 Years Post-Treatment (Mean ± SD)	Mean Difference (95% CI)	Statistical Analysis
Physical Health (PH)	10.30 ± 2.76	17.30 ± 0.91	7.00 (5.75–8.24)	*t*(29) = 11.54 *p* < 0.001 Cohen’s *d* = 3.64
Bodily Image & Appearance (BIA)	10.77 ± 2.94	17.23 ± 0.97	7.16 (5.98–8.34)	*t*(29) = 12.40 *p* < 0.001 Cohen’s *d* = 3.30
Level of Independence (LOI)	8.87 ± 2.28	15.23 ± 1.43	6.36 (5.52–7.21)	*t*(29) = 15.38 *p* < 0.001 Cohen’s *d* = 3.26
Social Relations (SR)	14.33 ± 1.58	16.80 ± 1.16	2.53 (2.04–3.02)	*t*(29) = 10.62 *p* < 0.001 Cohen’s *d* = 1.76
Financial Resources (FR)	13.20 ± 1.58	15.90 ± 1.24	2.70 (2.27–3.12)	*t*(29) = 12.86 *p* < 0.001 Cohen’s *d* = 1.83
New Info & Skills (OFIS)	16.43 ± 1.30	17.30 ± 1.15	0.93 (0.49–1.37)	*t*(29) = 4.36 *p* < 0.001 Cohen’s *d* = 0.75

Paired-samples *t*-test.

## Data Availability

The original contributions presented in this study are included in the Supplementary Material. Further inquiries can be directed to the corresponding author (M.R.).

## Supplementary Materials

The following supporting information can be downloaded at: https://www.mdpi.com/article/10.3390/healthcare14152357/s1, Table S1: Demographic distribution of respondents by age and sex; Table S2: Body mass index (BMI) of respondents by sex.

## Abbreviations

The following abbreviations are used in this manuscript:

LBP	Low Back Pain
RVT	Rhythmical Vibration Therapy
TMM	Traditional Mongolian Therapy
RMQ	Roland–Morris Disability Questionnaire
WHOQoL	World Health Organization’s Quality of Life Questionnaire
