# Supplementary material for: Traditional Mongolian Rhythmical Vibration Therapy for Low Back Pain: Acute Mechanisms and Three-Year Sustainability
Source: Healthcare (Basel). 2026 Aug 3;14(15):2357. doi: 10.3390/healthcare14152357 (PMC13464597; doi:10.3390/healthcare14152357)
Supplement: Supplementary file 1 [file healthcare-14-02357-s001.zip › healthcare-4341896-supplementary.pdf]

Table S1. Demographic distribution of respondents by age and sex

Age group	Female (n, %)	Male (n, %)	Total (n, %)
>20	15 (15%)	9 (9%)	24 (12.0%)
21 – 25	3 (3%)	6 (6%)	9 (4.5%)
26 – 30	6 (6%)	5 (5%)	11 (5.5%)
31 – 35	12 (12%)	16 (16%)	28 (14.0%)
36 – 40	13 (13%)	22 (22%)	35 (17.5%)
41 – 45	18 (18%)	18 (18%)	36 (18.0%)
46 – 50	14 (14%)	9 (9%)	23 (11.5%)
51 – 55	6 (6%)	7 (7%)	13 (6.5%)
56 – 60	6 (6%)	4 (4%)	10 (5.0%)
61>	7 (7%)	4 (4%)	11 (5.5%)
Total	100 (100%)	100 (100%)	200 (100%)
Average age	39.7 ± 16.7	38.7 ± 11.4	39.2 ± 12.6
95% CI	36.9 – 42.3	36.5 – 41.0	37.4 – 40.9

Table S2. Body mass index (BMI) of respondents by sex

	BMI		
	**Mean**	**95% CI**	***p-value**
Male	27.1 ± 4.4	26.5 – 28.3	
Female	26.4 ± 4.6	25.5 – 27.3	
Total	26.9 ± 4.5	26.3 – 27.5	0.023

*One-sample T-test
